# Assessing the protective effect of *Crassocephalum vitellinum* against Rifampicin-induced hepatotoxicity in Wistar rats

**DOI:** 10.4314/ahs.v22i1.43

**Published:** 2022-03

**Authors:** Kenedy Kiyimba, Emmanuel Tiyo Ayikobua, Daniel Chans Mwandah, Samuel Baker Obakiro

**Affiliations:** 1 Department of Pharmacology and Therapeutics, Faculty of health sciences, Busitema University; 2 Department of Physiology, School of Health Sciences, Soroti University; 3 Department of Pharmacology and Toxicology, School of Pharmacy, Kampala International Univrsity, Western Campus

**Keywords:** *Crassocephalum vitellinum*, antitubercular drugs, drug-induced hepatotoxicity, hepatoprotective agents

## Abstract

**Background:**

*Crassocephalum vitellinum* is widely used by traditional medical practitioners and local people in East Africa to manage a large number of ailments including hepatitis 1. However, its hepatoprotective effects had not been evaluated prior to this study. The aim of this study was to assess the effect of an ethanolic leaf extract of *Crassocephalum vitellinum* against rifampicin-induced liver toxicity in Wistar rats.

**Methods:**

Increasing doses of an ethanolic leaf extract of *C. vitellinum* were administered to Wistar rats daily for 35 days, together with rifampicin given orally as suspension. After the treatment period, Assessment of hepatoprotective activity was done by analysis of serum levels of biochemical and histopathological effects on the liver.

**Results:**

The results showed that administration of *C. vitellinum* extract significantly prevented drug- induced increase in serum levels of liver biomarker enzymes and also decreased the hepatocellular necrosis and inflammatory cells infiltration.

**Conclusion:**

The plant extract loweres the liver biomarker enzymes (ALT, ALP, AST) and preserves the histomorphology of the hepatocytes which is suggestive that the plant possess hepatoprotective properties.

## Introduction

Tuberculosis (TB) is among the leading killer infectious diseases in Uganda and the world at large 2. At a global level in 2017, TB caused an estimated 1.3 million deaths (range, 1.2–1.4 million) among HIV-negative people and there were an additional 300 000 deaths from TB (range, 266 000–335 000) among HIV-positive people. Globally, the best estimate is that 10.0 million people (range, 9.0–11.1 million) developed TB disease in 2017: 5.8 million men, 3.2 million women and 1.0 million children^3^.

The first line anti-tubercular drugs which include rifampicin, isoniazid, ethambutol and pyrazinamide have played a very significant therapeutic role in the management of the non-resistant form of TB. Although this therapy is very effective in managing the non- resistant TB strains, it has been associated with multiple adverse drug reactions which are life threatening. These include hepatitis, peripheral neuropathy, gastro intestinal disorders such as nausea, anorexia and vomiting 4.

Among the four first line anti-TB drugs, isoniazid (INH) is the most likely to cause drug induced liver toxicity. The incidence of hepatotoxicity in decreasing order is INH>PZA>RIF 5.

Fixed dose combinations such as Forecox®, Rimactazid ®, and Rimcure® are currently used in the management of TB and these may contain two or three or even four first line anti -tubercular agents formulated into a single tablet which increases the potential to cause adverse effects since such effects are sometimes potentiated by multiple drug regimens 5.

Thus, though INH, rifampicin and pyrazinamide each in itself are potentially hepatotoxic, when given in combination, their toxic effect is enhanced 6. It has also been reported that nearly 20% of patients treated with the standard four- drug regimen, show asymptomatic increase in aspartate aminotransferase concentration and that it usually occurs during the first 3 months of treatment 7. The mechanism of liver damage of these agents is not well described but many studies have linked it to oxidative stress that the metabolites (free radicals in nature) of the drugs cause in the body. These reactive oxygen species can damage DNA and lead to oxidation of lipids and proteins in hepatocytes hence hepatotoxicity^8^.

There exist controversies on how to manage anti-tubercular drug induced hepatotoxicity in different countries. From the view point of the WHO, it is recommended that when hepatotoxicity develops, the patient should be discontinued from the medication for some time until when the adverse effect is managed and then therapy continues 3.

This may lead to prolonging of treatment, drug resistance, and treatment failure. It may also increase morbidity and mortality of disease 9. Additionally, hepatotoxicity affects the detoxification role of the liver, which may result into accumulation of highly toxic substances and metabolites in the body resulting into death^4^. Hence the need for a preventive remedy for this drug induced liver toxicity.

*Crassocephalum vitellinum* (Benth) S. Moore (Asteraceae), a flowering herb from Africa is commonly found in countries like Burundi, Cameroon, Kenya, Rwanda, Uganda, Tanzania, Zambia, DRC 10. This plant is widely used by traditional medical practitioners and local people in East Africa to manage ailments such as hepatitis, woman sterility, strong fever, dysmenorrhea, diabetes, facilitating the deliverance of placenta, constipation and kids' diseases 1,11.

The leaf extract is used to manage eye infections, boils, anemia, poisoning, diarrhea and fresh wounds in Uganda 12, the aerial parts of *C. vitellinum* are boiled with water and the resulting decoction is taken regularly for management of peptic ulcers 13. Reports from other areas show that in Kenya, the plant is used for the treatment of stomach complications, malaria and mouth infections in children 14. In Southern Rwanda C.vitellinum is used as a hepatoprotective remedy which has eventually been linked to its antioxidant properties 1.

A number of studies have scientifically validated the efficacy of the crude ethanol extract of Crassocephalum vittellinum and have shown that the plant has antimalarial, antibacterial, cytoprotective, anti-inflammatory and hepatoprotective properties 15. However, prior to this study, there was hardly any study aimed at assessing the hepatoprotective activity of *C. vittellinum* against antitubercular drugs, yet the indigenous people have the perceived efficacy about it and they use the plant aqueous extracts in decoctions with other plant extracts to manage a number of ailments including hepatitis.

Rifampicin has been listed to be one of the backbones of TB pharmacotherapy 16, however that comes along with a risk of hepatotoxicity^17^, therefore this study sought to evaluate the hepatoprotective potential of *C.vitellinum* ethanolic extract against rifampicin induced hepatotoxicity so as to increase the confidence in extrapolating efficacy of the plant to humans, particularly with reference to its use as a herbal medicine.

## Materials and methods

### Plant collection and authentication

The leaves of Crassocephalum vitellinum were collected from Bushenyi District, Uganda. This area was chosen because of the abundance and widespread use of the plant in the management of hepatitis in this region. A voucher specimen (KK 001) was prepared and deposited at the herbarium of Botany department of Mbarara University of science and technology for correct botanical identification of the plant species by a taxonomist.

### Extraction of plant materials

The collected samples were dried under a shade to avoid direct sunshine that could degrade some of the compounds in the plants. They were put on a dry cemented bench in the Pharmacology Animal house at KIU, Western campus, and changed daily to prevent fungal attack until a constant weight was obtained. After drying, the leaves were pounded by use of a mortar and pestle, sieved to obtain a powder, weighed using an analytical scale (model ME104) and packed in clean labeled bottles until extraction.

The dry powder was extracted using cold maceration technique. A total crude ethanol extract was prepared by soaking 512 g of the powder in 1000 ml 99% W/V of ethanol (AR grade, batch number A-1007, Parchem) for seven days with occasional shaking to facilitate extraction. The extracts were strained using cotton wool and then filtered through a filter funnel using Whatman's filter paper No.1, and then the filtrate concentrated under reduced pressure using a Rotary Evaporator (model R-205V, SIGMA-ALDRICH) at 40°C.

The percentage yield of 7.08 % was obtained.

### Experimental design and animals

#### Experimental animals

Wistar albino rats (Rattus norvegicus) of both sexes weighing about (150–200g), aged 8–12 weeks were purchased from the Pharmacology Laboratory animal house of Kampala International University, Western Campus. These were acclimatized for 2 weeks before commencement of the study.

The animals were handled in conformity with guidelines for use of animals in research which included keeping them in standard cages under standard temperature conditions (23 ± 20 C), and illumination (12h light/dark cycle) while feeding them with agrinnovate rat pellets which is a standard diet for rats and water *ad libitum*^18^.

#### Experimental design

A total of 36 wistar albino rats (18 males, 18 females) were randomized among 6 treatment groups (6 rats/group); the dose concentrations were based on the acute toxicity study performed by 19.

Group I- 5 ml/kg normal saline (Healthy control)

Group II- RIF-(10mg/kg) dissolved in 5mls of normal saline (Negative control)

Group III- RIF followed by 200mg/kg of *Crassocephalum vitellinum* leaf extract 45 minutes later

Group IV - RIF followed by 400mg/kg of *Crassocephalum vitellinum* leaf extract 45 minutes later

Group V - RIF followed by 800mg/kg of *Crassocephalum vitellinum* leaf extract 45 minutes later

Group VI - RIF followed by 100mg/kg of silymarin (Positive control) 45 minutes later

The rats were orally administered with both the drugs and extract using a gastro intestinal tube daily for 35 days. During the experiment the animals were fed on rat pellets and given free access to water. Male rats were kept separate from females to prevent mating.

### Assessment of Hepatoprotective activity

#### Biochemical evaluation

After 35 days of administration, all the animals were anesthetized using ketamine (50mg/kg Body weight)^20^, sacrificed and blood collected into non heparinized vacutainers (4mls) for biochemical analysis. It was allowed to coagulate, and then centrifuged at 3000 rpm for 5 minutes to obtain serum.

The serum was then analyzed using an automated clinical chemistry analyzer (COBAS E 6000) to determine the serum levels of aspartate aminotransferase (AST) (U/L), alanine aminotransferase (ALT) (U/L) and alkaline phosphatase (ALP) (U/L) 21.

#### Histopathological evaluation

The animals were dissected to obtain the liver for histopathological evaluation. Gross examination of observable changes and weight of the liver were performed. The tissues obtained were isolated, fixed in 10% buffered formalin labeled bottles and transported to College of Veterinary Medicine, Animal Resources and Bio-Security (CoVAB) for further analysis. Tissue processing was done using an automated tissue processer. This involved dehydrating the fixed tissues placed in tissue cassettes with graded alcohol concentration (70%, 80%, 90% and 96%) respectively. The tissues were then removed after dehydration and then placed into xylene solution baths to clear off the alcohol and then facilitate molten wax impregnation. The tissues were then sectioned by use of Rotary microtome (Leica RM2245) (at 5um thickness), and then stained with hematoxylin and eosin (H & E) stain.

Slides were prepared and then examined by a research microscope connected to computerized image analyzer software (OLYMPUSstream J909D). Images were captured and then examined for histopathological changes by three independent pathologists who were not aware of the biochemical data to avoid bias 22.

### Statistical analysis

All numerical data generated were expressed as mean ± SEM (standard error of the mean and statistical comparison was performed using one-way analysis of variance (ANOVA), followed by the Tukey's post-hoc test, and values with P<0.05 were considered statistically significant 23. Statistical analysis was done using SPSS Version 6. Histopathological data was analyzed by a pathologist who wasn't aware of any treatment

### Ethical considerations

This study was performed in accordance with the National Institute of Health guidelines of animal research and guidance 18. Ethical approval was sought from (Institutional Research and Ethics committee) IREC KIU and an ethical approval number IREC369KIU was given. Intellectual property rights was observed and cost benefit sharing to the community.

## Results

### Effect of the crude ethanol leaf extract of *Crassocephalum vitellinum* on the biomarkers of antitubercular drug- induced liver toxicity in wistar rats

Biochemical results indicated significant changes and variations in the serum concentrations of liver enzymes including alanine aminotransferase (ALT), alkaline phosphatase (ALP), aspartate aminotransferase (ASP) in between the control group and the other groups that received the rifampicin together with the plant extract Rifampicin induced a significant hepatic injury in the animals and hence significant changes in the serum levels of the biomarker enzymes, AST, ALT, ALP were observed basing on the comparisons between control group I and group II(I+R+P) animals which is indicative of its hepatotoxic nature.

Treatment with the ethanolic leaf extract of *Crassocephalum vittellinum* in group III (200mg/kg), IV (400mg/kg), V(800mg/kg) respectively demonstrated a dose dependent degree of hepatoprotectivity against rifampicin as indicated by the percentage protection that the different doses of the extract demonstrated per marker enzyme, AST (11.86%, 47.78%, 68.23%), ALT (26.28%,48.05%, 72.73%), ALP (16.39%, 52.08%,79.12%) respectively as compared to group II that received only rifampicin). This indicates a dose dependent effect of the extract whereby animals that received the highest dose of the extract (800mg/kg) demonstrated a maximum percentage protection on AST (68.23%), ALT (72.73%), ALP (79.12%) and this is comparable with the percentage protection demonstrated by a standard hepatoprotective agent silymarin as follows AST (70.65%), ALT (76.74%), ALP (92.52%).

### Effect of the crude ethanol leaf extract of *Crassocephalum vitellinum* co-administered with rifampicin on the histomorphology of the liver in wistar rats

The histomorphological observations of the liver slices of the rats in all groups indicated significant variations between the control group and the treatment groups. Histopathology of group I (normal control) rats showed central vein surrounded by hepatic cord of cells and this is significant of normal liver tissue architecture. Rifampicin in group II animals induced a significant hepatic necrosis and this is evidenced by the large degree of liver cell necrosis with inflammatory collections and loss of cellular boundaries and loss of a central vein which is significant of liver hepatotoxicity (Figure 1).

**Figure:**
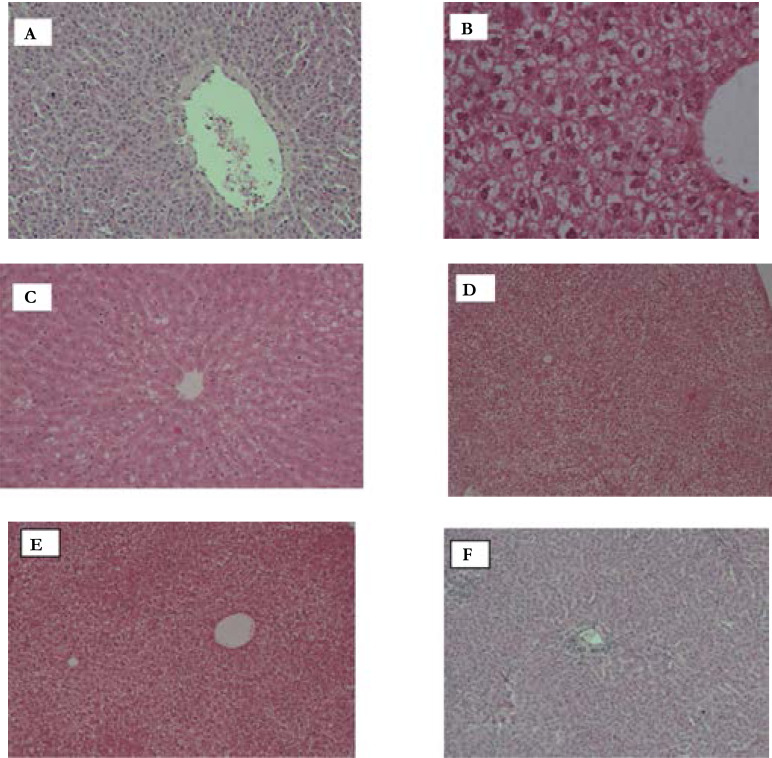
^A^ Liver section of normal control rat showing central vein surrounded by hepatic cord of cells (normal architecture), ^B^ Liver section of rifampicin treated rats showing massive fatty changes, focal necrosis with portal inflammation and loss of cellular boundaries, ^C^ Liver section of rats RIF and 200 mg/kg of CVITT showing mild focal necrosis with sinusoidal dilatation, ^D^ Micro views of Liver tissue section of rats treated with RIF and 400 mg/kg of CVITT showing, absence of necrosis, slight congestion in central vein and less inflammatory cells, ^E^ Micro views of Liver tissue section of rats treated with RIF and 800 mg/kg of C.vitellinum showing regeneration of hepatocytes around central vein toward near normal ***liver architecture,*** Key: CVITT- Crassocephalum vittellinum, RIF: rifampicin CV: central vein

The liver sections of all the groups of rats that were treated with the ethanolic extract of *Crassocephalum vitellinum* leaf extract showed absence of cell necrosis, but with minimal inflammatory conditions.

A dose dependent effect of the extract was observed, the groups that received highest dose of the extract (800mg/kg) and a standard hepatoprotective agent silymarin showed regeneration of hepatocytes around central vein with near normal liver architecture possessing higher hepatoprotective action as compared to the other two groups that received the lower doses of the extract i.e. 200mg/kg and 400mg/kg respectively.

These histomorphological results strongly support the biochemical results of the marker enzymes and its indicative that the ethanolic leaf extract of *Crassocephalum vitellinum* possess hepatoprotective properties against rifampicin induced liver toxicity.

## Discussion

The study sought to evaluate the protective effect of an ethanolic leaf extract of *C.vittellinum* plant extract against antitubercular drug induced hepatotoxicity. We used the wistar rat animal model to evaluate this effect, the animals were co administered with rifampicin together with an ethanolic leaf extract of *C.vitellinum* for 35 days and after the treatment period, and analysis of biochemical and histopathological parameters was carried out. Our findings demonstrated that the coadministration of the plant extract with the drugs ameliorated the hepatotoxic effects of the later.

Studies have indicated that the major challenge to TB pharmacotherapy is the hepatotoxic nature of the regimen used in TB management 24. Approximately 10% of the patients on isoniazid based therapy have developed hepatotoxicity 25. In a study to assess the causal association of isoniazid preventive therapy and hepatotoxicity and identify possible risk factors in patients on Highly Active Anti-retroviral Therapy (HAART), a total of 31 of cases of hepatotoxicity related to isoniazid preventive therapy were detected. Majority (80.6%) of the cases were marked as “serious” due to life-threatening situation (n = 15), hospitalization (n = 6), and death (n = 4) 26.

The mechanisms of the hepatotoxic nature of the antitubercular agents are unclear however, it has been attributed to the formation of oxidative reactive free radicals after the drugs have been metabolised by the cytochrome P-450 enzyme system 27. Hepatic N-acetyltransferase2 metabolize INH to acetylisoniazid which is then hydrolyzed to acetylhydrazine, a metabolite that is oxidized by cytochrome P450 to form some hepatotoxic intermediates, a reason as to why elevation of hepatic CYP 450 enzymes (CYP2E 1) has been implicated in the INH hepatotoxicity 28. A combination of INH and RIF has been associated with synergistic hepatotoxicity amongst patients which is a major drawback in the treatment of the infection since the two agents are the backbone of the therapy 29.

Rifampicin is an Antimycobacterial agent that belongs to the class of Rifamycins, it's metabolised in the liver by desacetylation to form desacetylrifamipicin, a metabolite that is microbiologically active and responsible for the antibacterial activity of the parent compound, a separate pathway of hydrolysis produces 3-formyl rifampicin 30.

These metabolites are less toxic to the liver however the hepatotoxic effect of the drug has been attributed to it being a cytochrome P450 enzyme system inducer (CYP3A4) via the hepatocyte PXR 31.

Our findings further confirm that the rifampicin induces serious hepatic tissue damage a result which agrees with an earlier study which evaluated the antihepatotoxic potential of Solanum xanthocarpum fruit extract against antitubercular drug-induced hepatopathy in experimental rodents 32.

Although the mechanism of hepatotoxicity of the drug seems unclear, oxidative stress caused by free radical metabolites of drugs has been strongly linked to this adverse drug reaction and a number of studies have demonstrated the presence of oxidative stress amongst patients with drug induced hepatotoxicity 33.

For redox homeostasis, the body ensures that there is a balance between prooxidants and antioxidants, through the antioxidant system that scavenges these reactive species, nonetheless, the exposure to antitubercular drugs induces the production of excessive reactive oxygen species which disrupt this homeostasis resulting into hepatotoxicity 8. Food and plants have been scientifically proven to have antioxidative phytochemicals and there concomitant use with hepatotoxic agents has been associated with reduced hepatotoxic effects of the agents 34.

Phytochemical analysis of crassocephalum vitellinum showed that the plant contains tannins, saponins, flavonoids and triterpenes 34. Tannins and flavonoids have been shown to be natural exogenous scavengers of free reactive oxygen species that are responsible for oxidative stress. Co-administration of this plant extract demonstrated *C.vittellinum* has the potential to ameliorate the hepatotoxic effects of rifampcin; this is attributed to the presence of the above phytochemicals that might have counteracted the toxic effects of the drug.

The extract demonstrated a dose dependent effect whereby animals that received the highest dose of the extract (800 mg/kg) demonstrated a maximum percentage protection on AST, ALT, and ALP which is comparable to the percentage protection demonstrated by a standard hepatoprotective agent silymarin. Such a result is suggestive of a high hepatoprotective activity as the dose of the extract is increased and further validates the ethno botanical claims by traditional medicine practitioners hence justifying its use in liver diseases in the different parts of East Africa.

Results of histopathological analysis strongly support the results from our investigations of the effects on biochemical indicators of hepatotoxicity. Rifampicin induced significant hepatic tissue necrosis which was observed in group II animals. Liver sections in this group showed massive fatty changes, focal necrosis with portal inflammation, and loss of cellular boundaries, and this is in line with results by 32. for signs of liver toxicity. Treatment with the ethanolic extract of *C. vittellinum* at different doses showed an increasing degree of protection against the toxic effects of the drug with the animals that received 200 mg/kg of the extract showing mild focal necrosis with sinusoidal dilatation, whereas the liver tissues in the preceding treatment group (group IV) showed absence of necrosis, slight congestion in central vein and less inflammatory cells. Rats that received the highest dose (800 mg/kg) of the extract showed a near to normal liver architecture-showing regeneration of hepatocytes around central vein, which was comparable to the animals that received a standard hepatoprotective agent, silymarin. This implies that *crassocephalum vitellinum* possess a good degree of hepatoprotective activity and hence may serve as a lead to a potential hepatoprotective agent.

Furthermore, it was noted that, although the extract showed hepatoprotective properties, it provides a maximal effect at a very high dose (800mg/kg). This may be attributed to the fact that this was a crude extract in which the content of active compounds could be low. Isolation of the active ingredients might increase the potency of the extract although, sometimes, there is loss of activity when the activity of the compounds in a crude mixture is a result of synergistic action 32.

## Study limitations

Our study did not assess other parameters like antioxidant activity, biochemical assays such as serum bilirubin, total protein, gamma-glutamyl transferase (ggt) which would further inform about the hepatoprotective potential of the ethanolic plant extract of C.vitellinum against rifampicin induced hepatotoxicity.

## Conclusions and Recommendations

On the basis of the findings our study showed that the ethanolic leaf extract of crassocephalum vitellinum dose dependently protected the animals from rifampicin induced hepatotoxicity.

Further studies on the antioxidant activity of the plant should be done. Isolation, characterization and structure elucidation of the hepatoprotective active compound and its mechanism of action should as well be determined.

## Figures and Tables

**Table 1:** The effect of *C. vitellinum* extract on liver biomarkers against anti-tubercular drug induced liver toxicity

GROUP	SERUM PARAMETER		
	AST(U/L)	ALT(U/L)	ALP(U/L)
(GROUP I) Healthy control	117 ± 1.36	50.95 ± 1.55	70.12 ± 1.72
RIF (GROUP II)	425.9 ± 4.45*	262.2 ± 1.92*	173.8 ± 2.27*
200mg/kg *C.vitellinum* (GROUP III)+ IRP	375.4 ± 1.98#	193.3 ± 1.18#	145.3 ± 1.33#
400mg/kg *C.vitellinum* (GROUP IV)+ IRP	222.4 ±0.91###	136.2 ±1.62###	121.7 ± 1.12 ###
800mg/kg *C.vitellinum* (GROUP V)+ IRP	135.3 ±1.09###	71.5 ± 1.92###	94.7 ± 0.57###
SILYMARIN(100mg/kg) GROUP VI+ IRP	125±1.81###	60.98 ±0.79###	81.28 ± 1.95###

Values are expressed as mean ± SEM

* P<0.001 compared with control group 1

# P<0.05

## P<0.01

### P<0.001 compared with group II (I+R+P), n=6,RIF-rifampicin, U/L-units/litre

Co-administration of antitubercular drugs with crassocephalum vitellinum extract dose dependently lowered the serum levels of AST, ALT and ALP

## Role of authors

KK was the principle investigator and conceptualized the study execution. DCM and ETA, SBO participated in proposal and manuscript development and research. All authors have read and approved the final manuscript
